# Involvement of reduced ENPP1 function in the pathogenesis of ossification of the posterior longitudinal ligament

**DOI:** 10.1093/jbmrpl/ziag157

**Published:** 2026-09-17

**Authors:** Soichiro Kimura, Koichiro Furukawa, Koki Irie, Natsuho Adachi, Yoshitomo Hoshino, Naoko Hidaka, Hajime Kato, Junya Miyahara, Yasushi Oshima, Toru Doi, Masaomi Nangaku, Noriko Makita, Sakae Tanaka, Taku Saito, Nobuaki Ito

**Affiliations:** Division of Nephrology and Endocrinology, The University of Tokyo Hospital, Bunkyo, Tokyo 113-8655, Japan; Osteoporosis Center, The University of Tokyo Hospital, Bunkyo, Tokyo 113-8655, Japan; Division of Nephrology and Endocrinology, The University of Tokyo Hospital, Bunkyo, Tokyo 113-8655, Japan; Osteoporosis Center, The University of Tokyo Hospital, Bunkyo, Tokyo 113-8655, Japan; Division of Nephrology and Endocrinology, The University of Tokyo Hospital, Bunkyo, Tokyo 113-8655, Japan; Osteoporosis Center, The University of Tokyo Hospital, Bunkyo, Tokyo 113-8655, Japan; Osteoporosis Center, The University of Tokyo Hospital, Bunkyo, Tokyo 113-8655, Japan; Department of Pediatrics, The University of Tokyo Hospital, Bunkyo, Tokyo 113-8655, Japan; Division of Nephrology and Endocrinology, The University of Tokyo Hospital, Bunkyo, Tokyo 113-8655, Japan; Osteoporosis Center, The University of Tokyo Hospital, Bunkyo, Tokyo 113-8655, Japan; Division of Nephrology and Endocrinology, The University of Tokyo Hospital, Bunkyo, Tokyo 113-8655, Japan; Osteoporosis Center, The University of Tokyo Hospital, Bunkyo, Tokyo 113-8655, Japan; Division of Nephrology and Endocrinology, The University of Tokyo Hospital, Bunkyo, Tokyo 113-8655, Japan; Osteoporosis Center, The University of Tokyo Hospital, Bunkyo, Tokyo 113-8655, Japan; Department of Pathology, Yale University School of Medicine, New Haven, CT 06510, United States; Department of Orthopaedic Surgery, The University of Tokyo Hospital, Bunkyo, Tokyo 113-8655, Japan; Department of Orthopaedic Surgery, The University of Tokyo Hospital, Bunkyo, Tokyo 113-8655, Japan; Department of Orthopaedic Surgery, Tokyo Women’s Medical University, Shinjuku, Tokyo 162-8666, Japan; Division of Nephrology and Endocrinology, The University of Tokyo Hospital, Bunkyo, Tokyo 113-8655, Japan; Division of Nephrology and Endocrinology, The University of Tokyo Hospital, Bunkyo, Tokyo 113-8655, Japan; Osteoporosis Center, The University of Tokyo Hospital, Bunkyo, Tokyo 113-8655, Japan; Department of Orthopaedic Surgery, The University of Tokyo Hospital, Bunkyo, Tokyo 113-8655, Japan; Osteoporosis Center, The University of Tokyo Hospital, Bunkyo, Tokyo 113-8655, Japan; Department of Orthopaedic Surgery, The University of Tokyo Hospital, Bunkyo, Tokyo 113-8655, Japan; Osteoporosis Center, The University of Tokyo Hospital, Bunkyo, Tokyo 113-8655, Japan; Division of Therapeutic Development for Intractable Bone Diseases, Graduate School of Medicine and Faculty of Medicine, The University of Tokyo, Bunkyo, Tokyo 113-8655, Japan

**Keywords:** ossification of the posterior longitudinal ligament, ENPP1, pyrophosphate, diffuse idiopathic skeletal hyperostosis, ectopic ossification, enthesopathy

## Abstract

Ossification of the posterior longitudinal ligament (OPLL) is an ectopic ossification disorder with incompletely understood pathophysiology. In a previously reported 50-patient OPLL cohort, ectonucleotide pyrophosphatase/phosphodiesterase 1 (*ENPP1*) exonic heterozygous variants were identified in 7 patients (14%), and plasma inorganic pyrophosphate (PPi), a biochemical marker reflecting ENPP1 function, was lower in patients with OPLL than in cervical osteoarthritis controls regardless of *ENPP1* variant status. However, the clinical significance of *ENPP1* variants and reduced PPi in OPLL, as well as the possible presence of non-genetic ENPP1 dysfunction, remains unclear. The study included the same cohort of 50 patients with OPLL as in our previous study, 7 of whom had *ENPP1* variants. Clinical, biochemical, and radiographic characteristics were compared according to *ENPP1* variant status and cohort median PPi level. Anti-ENPP1 autoantibodies were evaluated in an exploratory analysis using a luciferase immunoprecipitation system (LIPS) assay in 29 patients with available serum samples. Patients with *ENPP1* variants showed higher serum FGF23 levels than those without variants (median 46 vs 36 pg/mL, *p* = .011) and tended to have lower plasma PPi levels (1477 vs 1693 nM, *p* = .052), suggesting partial impairment of ENPP1 function. However, radiographic ossification indices, diffuse idiopathic skeletal hyperostosis (DISH), and surgical characteristics did not differ by variant status. In contrast, DISH was more frequent in the lower-PPi group than in the higher-PPi group (72% vs 44%, *p* = .045). No apparent anti-ENPP1 autoantibodies were detected using the LIPS assay. Ectonucleotide pyrophosphatase/phosphodiesterase 1 exonic heterozygous variants may contribute to OPLL through partial ENPP1 dysfunction. More broadly, reduced ENPP1 function itself, regardless of variant status, may be involved in ectopic ligament ossification in OPLL, although anti-ENPP1 autoantibodies were not detected in the current study. The broader involvement of ENPP1 dysfunction in OPLL may indicate a wider population that could potentially benefit from future ENPP1 replacement therapy.

## Graphical Abstract

**Graphical Abstract:**
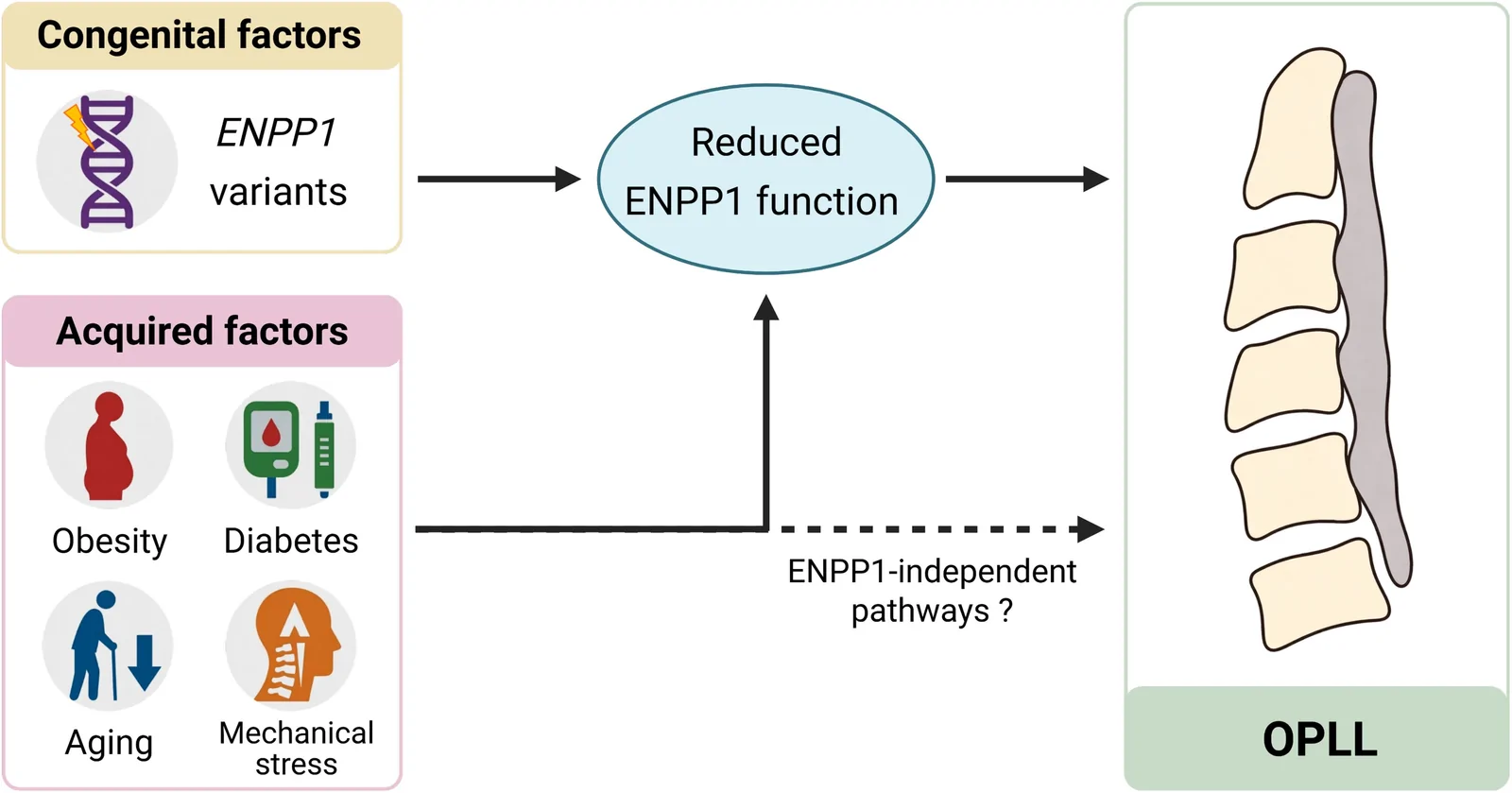

## Introduction

Ossification of the posterior longitudinal ligament (OPLL) is a disorder characterized by ectopic ossification of the spinal posterior longitudinal ligament. As the disease progresses, it can cause spinal canal stenosis and lead to neurological symptoms such as myelopathy and radiculopathy. Ossification of the posterior longitudinal ligament exhibits geographic and ethnic variation in prevalence, with reported rates of approximately 1.9%-4.3% in Japan, 4.6% in South Korea, and 4.1% in China, whereas the prevalence is lower in Caucasian populations in North America at approximately 1.3%.^1–4^ Furthermore, even within the same North American population, the prevalence has been reported to be higher in Asian Americans (4.8%), suggesting a contribution of genetic background to the development of OPLL.

Diffuse idiopathic skeletal hyperostosis (DISH) is a condition characterized by ossification and calcification of spinal ligaments and peripheral entheses. Diffuse idiopathic skeletal hyperostosis frequently coexists with OPLL and shares several radiological and clinical features with it, suggesting that these conditions may involve partially overlapping mechanisms of enthesopathic ossification.^5^^,^^6^

The development of OPLL is associated with metabolic factors, such as aging, obesity, and type 2 diabetes mellitus, as well as environmental factors including mechanical stress on spinal ligaments. However, the precise pathophysiological mechanisms underlying OPLL remain incompletely understood.^7^^,^^8^

Meanwhile, ectonucleotide pyrophosphatase/phosphodiesterase 1 (ENPP1) has been suggested to play a role in the pathogenesis of ligament ossification. Ectonucleotide pyrophosphatase/phosphodiesterase 1 is an enzyme that hydrolyzes extracellular adenosine triphosphate (ATP) to generate inorganic pyrophosphate (PPi), and plasma PPi levels are considered a biochemical marker reflecting ENPP1 function.^9^

The ENPP1-deficient tiptoe walking mouse is a well-established animal model characterized by ossification of spinal ligaments, supporting a link between ENPP1 dysfunction and ligament ossification.^10^ In humans, biallelic loss-of-function variants in *ENPP1* are known to cause generalized arterial calcification of infancy and autosomal recessive hypophosphatemic rickets type 2 (ARHR2). Notably, some patients with these conditions have also been reported to develop severe spinal ligament ossification, particularly OPLL.^11^^,^^12^ Furthermore, in recent years, heterozygous exonic variants of *ENPP1* have been reported in cases of spinal ligament ossification, suggesting that a partial reduction in ENPP1 activity may contribute to the development of spinal ligament ossification.^13^

In a recently reported cohort of 50 patients with OPLL, *ENPP1* exonic heterozygous variants were identified in 7 patients (14%). Moreover, plasma PPi levels were lower in patients with OPLL than in those with cervical osteoarthritis (OA), regardless of *ENPP1* variant status.^14^ This finding suggests that ENPP1 function may be partially reduced even in patients without identifiable genetic variants. However, the detailed clinical characteristics of patients with *ENPP1* variants, the clinical significance of reduced PPi levels in OPLL, and the possibility of ENPP1 dysfunction driven by factors other than genetic variants remain insufficiently investigated.

In this study, using the same cohort, we compared the clinical characteristics of patients with OPLL according to *ENPP1* variant status to assess the phenotypic impact of *ENPP1* variants. We further examined the relationship between plasma PPi levels and clinical severity to evaluate whether the degree of ENPP1 dysfunction is associated with disease manifestations.

Furthermore, because OPLL is an acquired disorder, acquired reduction of ENPP1 function may contribute to its pathogenesis. In particular, inhibitory autoantibodies against ENPP1 could represent one such factor. The luciferase immunoprecipitation system (LIPS) assay is a highly sensitive immunoprecipitation method for detecting autoantibodies.^15^^,^^16^ More recently, application of this technique has shown that a subset of previously unexplained osteomalacia without detectable FGF23-producing tumors is mediated by autoantibodies against phosphate regulating endopeptidase X-linked, and this condition has been reported as autoimmune osteomalacia.^17^ Given the possibility that a similar acquired autoimmune mechanism may underlie OPLL, the presence of anti-ENPP1 autoantibodies was exploratorily assessed using a LIPS assay in the present study.

## Materials and methods

### Participants

Between January 1, 2021, and January 31, 2023, consecutive patients who were evaluated for surgical treatment of OPLL at the Department of Orthopaedic Surgery and Spinal Surgery, The University of Tokyo, were included in this study (*n* = 50). Exon sequencing of *ENPP1* was performed in the 50 patients with OPLL.^14^

This study was conducted in accordance with the Declaration of Helsinki and was approved by the Ethics Committee of The University of Tokyo Hospital (approval number: 11221). Written informed consent was obtained from all participants.

### Biochemical measurements and clinical data collection

In patients with OPLL, plasma PPi levels, a biomarker reflecting ENPP1 function, were measured using an ATP sulfurylase-based assay. Inorganic pyrophosphate levels were measured in the control group using the same method.^13^ Blood samples for plasma PPi measurement were generally collected in the morning in the fasting state, with patients resting before venipuncture. In addition, the following laboratory parameters were evaluated in patients with OPLL. HbA1c was measured using HLC-723G11 (Tosoh). Inorganic phosphate (Pi), albumin-adjusted calcium, alkaline phosphatase, and C-reactive protein (CRP) were measured using LABOSPECT008 (Hitachi). Intact parathyroid hormone was measured using AIA-CL2400 (Tosoh). Serum intact FGF23 was measured using Determinar CL FGF23, a chemiluminescent enzyme immunoassay (Canon Medical Diagnostics Corporation). For the exploratory analysis of inflammation, the CRP value obtained on the same day as plasma PPi sampling was used when available; otherwise, the preoperative CRP value closest to the PPi sampling date was selected. CRP data were available for 49 of the 50 patients.

Radiographic findings were assessed using the ossification of the anterior longitudinal ligament index (OA-index), OPLL index (OP-index), ossification of the yellow ligament index (OY-index), and the total ossification index (OS-index), defined as the sum of these indices. The presence of DISH was also evaluated. These radiographic assessments were independently performed by 2 board-certified orthopedic surgeons with clinical experience in spinal disorders.

### Anti-ENPP1 autoantibody analysis

To evaluate the presence of autoantibodies against ENPP1, a LIPS assay was performed. The LIPS analysis included serum samples from 29 patients with OPLL: 20 from the original cohort of 50 patients who underwent genetic analysis and 9 from additional patients enrolled later. All 20 patients from the original cohort had undergone both *ENPP1* genotyping and plasma PPi measurement. Among the 9 additional patients, *ENPP1* genotyping results were available for 6 patients, all of whom were negative for *ENPP1* exonic variants, and plasma PPi measurements were available for 2 patients. Overall, *ENPP1* genotyping and plasma PPi data were available for 26 and 22 of the 29 patients, respectively. The LIPS cohort included 8 patients who were negative for *ENPP1* exonic variants and had plasma PPi levels below the median of the original cohort. As negative controls, serum samples from 2 patients each with tumor-induced osteomalacia, Cushing’s disease, hypophosphatasia, Graves’ disease, and hypoparathyroidism were used. As a positive control, a commercially available anti-ENPP1 antibody (Biorbyt) was used. In addition, a buffer-only condition without patient serum was included for baseline luminescence measurements. Full-length ENPP1 constructs fused with NanoLuc luciferase (ENPP1-NanoLuc and NanoLuc-ENPP1) were generated and expressed in COS-7 cells, and the resulting cell lysates were used as antigens. Patient serum samples were incubated with Protein A/G magnetic beads (Dynabeads Protein A and G, Invitrogen), followed by immunoprecipitation with the antigen lysates. Luminescence was measured using Nano-Glo Luciferase Assay Substrate (Promega), and signals were detected with a GloMax Discovery Multimode Microplate Reader (Promega) and analyzed as relative luminescence units. Detailed methods are described in the supplementary methods.

### Statistical analysis

Continuous variables were expressed as median (range) and compared between groups using the Mann–Whitney U test. Categorical variables were expressed as percentage (number) and compared using Pearson’s chi-square test. A 2-sided *p*-value <.05 was considered statistically significant. Patients with OPLL were additionally stratified into 2 groups according to the median plasma PPi level for exploratory group comparisons. For multivariable analysis, plasma PPi levels were analyzed using analysis of covariance (ANCOVA), with *ENPP1* variant status as a fixed factor and age, BMI, and HbA1c as covariates. To examine the association between plasma PPi levels and DISH without dichotomizing PPi, binary logistic regression analyses were performed with the presence of DISH as the dependent variable and plasma PPi level as a continuous independent variable. Odds ratios were expressed per 100-nM increase in plasma PPi. Both an unadjusted model and a multivariable model adjusted for HbA1c and serum Pi were evaluated. For the exploratory CRP analyses, serum CRP levels were compared according to *ENPP1* variant status and plasma PPi group using the Mann–Whitney U test, and the association between serum CRP and plasma PPi levels and the associations between serum CRP and each radiographic ossification index (OA-index, OP-index, OY-index, and OS-index) were assessed using Spearman’s rank correlation coefficient. Correlations between serum CRP and the radiographic ossification indices were also examined separately according to *ENPP1* variant status. All statistical analyses were performed using JMP version 18 (SAS Institute Inc.).

## Results

A total of 50 patients with OPLL were included in this study. Among them, 1 patient (Patient 2) did not undergo surgery because conservative management was chosen after the initial evaluation.

Ectonucleotide pyrophosphatase/phosphodiesterase 1 heterozygous variants were identified in 7 of the 50 patients (14%) (Table S1).^14^ Clinical characteristics were compared between patients with and without ENPP1 heterozygous variants (Table 1). Patients with *ENPP1* variants tended to have a higher frequency of a family history of OPLL (29% vs 7%, *p* = .077). In addition, serum FGF23 levels were significantly higher in patients with *ENPP1* variants than in those without variants (median 46 vs 36 pg/mL, *p* = .011). In contrast, plasma PPi levels tended to be lower in patients with *ENPP1* variants, although the difference did not reach statistical significance (median 1477 vs 1693 nM, *p* = .052). After adjustment for age, BMI, and HbA1c using ANCOVA, *ENPP1* variant status was not significantly associated with plasma PPi levels. The adjusted least-squares mean plasma PPi levels were 1519 nM (SE, 168) in the variant-positive group and 1805 nM (SE, 67) in the variant-negative group (*p* = .123). Regarding clinical symptoms, gait disturbance and muscle weakness were less frequently observed in patients with *ENPP1* variants compared with those without variants (43% vs 79%, *p* = .043; and 14% vs 74%, *p* = .002, respectively). No significant differences were observed between the 2 groups in radiographic ossification indices, the presence of DISH, or surgical characteristics.

**Table 1:** Clinical characteristics of OPLL patients with or without *ENPP1* heterozygous variants.

	Variant positive (*n* = 7)	Variant negative (*n* = 43)	*p*
**Patient characteristics**			
**Family history of OPLL, % (*n*)**	29 (2)	7 (3)	.077
**Age at diagnosis, yr**	66 (35-80)	60 (38-84)	.644
**BMI**	23.1 (21.2-38.6)	25.7 (16.4-38.6)	.801
**Symptoms**			
**Pain, % (*n*)**	29 (2)	35 (15)	.744
**Paresthesia, % (*n*)**	100 (7)	84 (36)	.250
**Hand clumsiness, % (*n*)**	43 (3)	58 (25)	.450
**Gait disturbance, % (*n*)**	43 (3)	79 (34)	**.043**
**Muscle weakness, % (*n*)**	14 (1)	74 (32)	**.002**
**Laboratory data**			
**HbA1c, %**	5.6 (5.3-6.2)	5.9 (4.9-8.3)	.268
**Pi, mg/dL**	3.2 (2.8-4.1)	3.2 (1.9-5.1)	.684
**cCa, mg/dL**	9.3 (8.8-9.9)	9.2 (8.5-10.1)	.384
**eGFR, mL/min/1.73 m**^**2**^	58 (44-78)	66 (39-109)	.121
**ALP, U/L**	82 (46-198)	79 (30-175)	.889
**CRP, mg/dL**^**a**^	0.04 (0.02-0.63)	0.07 (0.02-0.73)	.863
**iPTH, pg/mL**	58 (22-125)	51 (23-142)	.442
**FGF23, pg/mL**	46 (36-102)	36 (14-86)	**.011**
**PPi, nM**	1477 (958-1915)	1693 (850-3012)	.052
**Imaging findings**			
**OA-index**	9 (0-17)	12 (0-30)	.484
**OP-index**	5 (3-12)	5 (0-36)	.978
**OY-index**	4 (2-13)	3 (0-14)	.200
**OS-index**	25 (7-29)	20 (3-60)	.812
**DISH, % (*n*)**	71 (5)	56 (24)	.438
**Surgical history**			
**Age at first surgery, yr**	69 (57-82)	63 (38-86)	.314
**Number of surgeries**	1 (0-1)	1 (1-3)	.057

Abbreviations: ALP, alkaline phosphatase; cCa, albumin-adjusted calcium; CRP, C-reactive protein; DISH, diffuse idiopathic skeletal hyperostosis; eGFR, estimated glomerular filtration rate; ENPP1, ectonucleotide pyrophosphatase/phosphodiesterase 1; iPTH, intact parathyroid hormone; OA-index, ossification of the anterior longitudinal ligament index; OP-index, ossification of the posterior longitudinal ligament index; OPLL, ossification of the posterior longitudinal ligament; OY-index, ossification of the yellow ligament index; OS-index, the total ossification index; Pi, inorganic phosphate; PPi, inorganic pyrophosphate.

Reference ranges were as follows: HbA1c, 4.9%-6.0%; Pi, 2.7-4.6 mg/dL; ALP, 38-113 U/L; iPTH, 10-65 pg/mL; and FGF23, 19.9-52.9 pg/mL.

^a^CRP data were available for 49 patients: 7 variant-positive and 42 variant-negative patients. Bold *p* values indicate statistically significant differences (*p* < 0.05).

Patients were stratified according to the median plasma PPi level in this cohort (1665 nM). Clinical characteristics of patients with OPLL in the above- and below-median PPi groups were compared in Table 2. The prevalence of DISH was significantly higher in the below-median PPi group than in the above-median group (72% vs 44%, *p* = .045). In terms of laboratory findings, HbA1c and serum Pi levels differed significantly between the 2 groups. No significant differences were observed in patient characteristics, clinical symptoms, radiographic ossification indices, or surgical characteristics. When plasma PPi was analyzed as a continuous variable, each 100-nM increase in plasma PPi was associated with numerically lower odds of DISH, although the association was not statistically significant (unadjusted OR, 0.91; 95% CI, 0.79-1.04; *p* = .157). The association remained nonsignificant after adjustment for HbA1c and serum Pi (adjusted OR, 0.91; 95% CI, 0.79-1.05; *p* = .202).

**Table 2:** Comparison of OPLL patients above and below the median plasma PPi level.

	Above median ^a^ (*n* = 25)	Below median (*n* = 25)	*p*
**Patient characteristics**			
**Family history of OPLL, % (*n*)**	12 (3)	8 (2)	.637
**Age at diagnosis, yr**	61 (38-84)	61 (35-83)	.969
**BMI**	26.2 (19.8-38.6)	24.9 (16.4-38.6)	.485
**Symptoms**			
**Pain, % (*n*)**	28 (7)	40 (10)	.371
**Paresthesia, % (*n*)**	80 (20)	92 (23)	.221
**Hand clumsiness, % (*n*)**	48 (12)	64 (16)	.255
**Gait disturbance, % (*n*)**	64 (16)	84 (21)	.107
**Muscle weakness, % (*n*)**	68 (17)	64 (16)	.765
**Laboratory data**			
**HbA1c, %**	6.0 (5.3-8.3)	5.6 (4.9-7.7)	**.029**
**Pi, mg/dL**	3.3 (2.7-5.1)	3.1 (1.9-4.1)	**.035**
**cCa, mg/dL**	9.3 (8.7-9.8)	9.2 (8.5-10.1)	.293
**eGFR, mL/min/1.73 m**^**2**^	73 (44-109)	64 (39-94)	.225
**ALP, U/L**	79 (30-114)	87 (42-198)	.116
**CRP, mg/dL**^**b**^	0.08 (0.02-0.48)	0.06 (0.02-0.73)	.623
**iPTH, pg/mL**	51 (24-125)	56 (22-142)	.351
**FGF23, pg/mL**	37 (14-62)	38 (19-102)	.404
**Imaging findings**			
**OA-index**	12 (0-20)	10 (0-30)	.690
**OP-index**	5 (1-19)	6 (0-36)	.762
**OY-index**	3 (0-14)	3 (0-13)	.584
**OS-index**	20 (3-49)	21 (4-60)	.655
**DISH, % (*n*)**	44 (11)	72 (18)	**.045**
**Surgical history**			
**Age at first surgery, yr**	65 (38-84)	64 (46-86)	.834
**Number of surgeries**	1 (1-2)	1 (0-3)	.805

Abbreviations: ALP, alkaline phosphatase; cCa, albumin-adjusted calcium; CRP, C-reactive protein; DISH, diffuse idiopathic skeletal hyperostosis; eGFR, estimated glomerular filtration rate; iPTH, intact PTH; OA-index, ossification of the anterior longitudinal ligament index; OP-index, ossification of the posterior longitudinal ligament index; OPLL, ossification of the posterior longitudinal ligament; OY-index, ossification of the yellow ligament index; OS-index, the total ossification index; Pi, inorganic phosphate; PPi, inorganic pyrophosphate.

^a^The median plasma PPi level in the entire cohort was 1665 nM.

^b^CRP data were available for 49 patients: 24 patients in the above-median PPi group and 25 patients in the below-median PPi group. Bold *p* values indicate statistically significant differences (*p* < 0.05).

C-reactive protein data were available for 49 patients. Serum CRP levels did not differ significantly between patients with and without *ENPP1* variants (median [range], 0.04 [0.02-0.63] vs 0.07 [0.02-0.73] mg/dL; *p* = .863) or between the lower- and higher-PPi groups (0.06 [0.02-0.73] vs 0.08 [0.02-0.48] mg/dL; *p* = .623). Serum CRP levels were not significantly correlated with plasma PPi levels (Spearman’s ρ = −0.026, *p* = .857). Serum CRP levels were also not significantly correlated with the OA-index (Spearman’s ρ = 0.177, *p* = .224), OP-index (ρ = −0.138, *p* = .343), OY-index (ρ = 0.085, *p* = .563), or OS-index (ρ = 0.092, *p* = .528). No significant correlations between serum CRP levels and any of the radiographic ossification indices were observed when the analyses were stratified by *ENPP1* variant status.

In the LIPS assay, the luminescence intensity increased by more than 100-fold in both antigen systems using ENPP1-NanoLuc and NanoLuc-ENPP1 when the commercial anti-ENPP1 antibody was used as a positive control, compared with buffer alone, confirming that the assay system functioned appropriately. In contrast, sera from all 29 patients with OPLL, including the 8 patients who were negative for *ENPP1* exonic variants and had plasma PPi levels below the median of the original cohort, yielded luminescence intensities as low as those of the negative controls in both antigen systems, indicating that no apparent anti-ENPP1 autoantibodies were detected (Figure 1).

**Figure 1:**
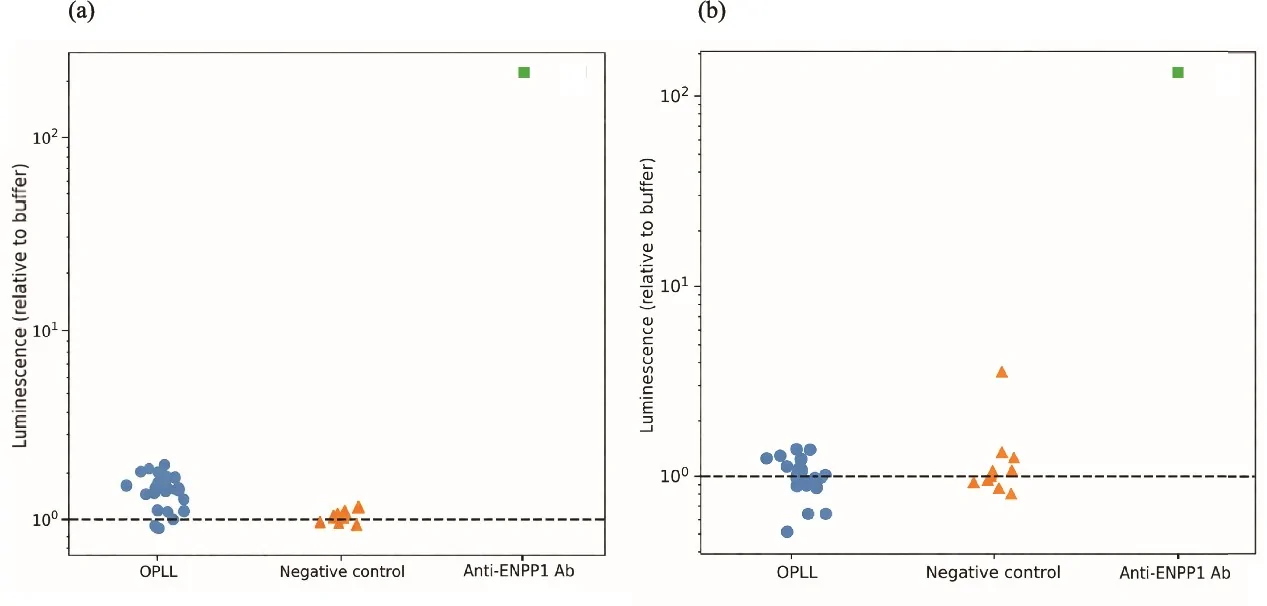
Detection of anti-ENPP1 autoantibodies in sera from patients with OPLL using a LIPS assay. Luminescence signals (relative to buffer) were measured in the LIPS assay using (A) ENPP1-NanoLuc and (B) NanoLuc-ENPP1 constructs. Serum samples from patients with OPLL (*n* = 29, circles), disease control sera (*n* = 10, triangles), and a commercially available anti-ENPP1 antibody as a positive control (squares) were analyzed. The anti-ENPP1 antibody produced more than a 100-fold increase in luminescence compared with buffer in both assay systems. In contrast, sera from patients with OPLL showed no increase in luminescence in either system and were comparable to those of negative control sera. ENPP1, ectonucleotide pyrophosphatase/phosphodiesterase 1; LIPS, luciferase immunoprecipitation system; OPLL, ossification of the posterior longitudinal ligament.

## Discussion

In this exploratory analysis of a previously reported OPLL cohort, patients with *ENPP1* variants had higher serum FGF23 levels and tended to have lower plasma PPi levels, whereas lower plasma PPi levels were associated with a higher prevalence of DISH regardless of variant status. No apparent anti-ENPP1 autoantibodies were detected. These findings suggest that reduced ENPP1 function may be relevant to spinal ligament ossification beyond *ENPP1* genotype alone, although the observed associations remain exploratory.

Although serum Pi levels were comparable between the 2 groups and remained within the normal to low-normal range, the higher serum FGF23 levels and trend toward lower plasma PPi levels in patients with *ENPP1* variants may reflect partial impairment of ENPP1 function. However, the absolute difference in median FGF23 levels was modest (46 vs 36 pg/mL), and both group medians were within the reference range (19.9-52.9 pg/mL); given the analytical and biological variability of FGF23 measurements, this finding should be interpreted cautiously as an exploratory biochemical signal. In addition, although the difference did not reach statistical significance, the proportion of patients with a family history of OPLL was higher in the variant-positive group than in the variant-negative group (29% vs 7%), suggesting that genetic background may still play a meaningful role in patients harboring *ENPP1* variants despite the limited sample size. Notably, gait disturbance and muscle weakness were less frequent in the variant-positive group, while no clear differences were observed in radiographic ossification indices, the prevalence of DISH, age at first surgery, or the number of surgeries. Given the small variant-positive subgroup, this counterintuitive pattern may partly reflect sampling variability or referral and selection effects; moreover, neurological severity depends on the anatomical level and degree of spinal cord compression rather than on overall ossification extent alone. In patients with biallelic ENPP1 deficiency, such as ARHR2, marked loss of ENPP1 function can be associated with severe enthesopathy and spinal ligament ossification. By contrast, the monoallelic *ENPP1* variants identified in the present cohort may exert a more modest functional effect and contribute to susceptibility to OPLL without necessarily resulting in greater clinical or radiographic severity than idiopathic OPLL.

When patients were stratified by plasma PPi level, the higher prevalence of DISH in the lower-PPi group, despite the absence of significant differences in radiographic ossification indices, raised the possibility that reduced ENPP1 function may be associated with ectopic ligament ossification irrespective of *ENPP1* variant status. Although the lower-PPi group also had lower HbA1c and serum Pi levels, the direction of the HbA1c difference argues against simple confounding by greater glycemic burden. In the continuous-variable analysis, the point estimate for the inverse association between plasma PPi and DISH was essentially unchanged after adjustment for HbA1c and serum Pi, although the association was not statistically significant. An independent case-control study reported that patients with DISH had higher serum FGF23 and lower serum Pi levels than matched controls, with serum FGF23 positively correlating with the extent of spinal ossification.^18^ In the present cohort, however, higher FGF23 levels without a corresponding difference in serum Pi were observed in patients with *ENPP1* variants, whereas lower serum Pi levels without a significant difference in FGF23 were observed in the lower-PPi subgroup. This discrepancy may partly reflect differences in the study populations and comparison strategies. The previous study directly compared patients with DISH and non-DISH controls, whereas the present study compared subgroups stratified by *ENPP1* variant status or plasma PPi level within a clinically more homogeneous cohort consisting of patients with OPLL evaluated for surgery. This within-cohort design may have contributed to less consistent biochemical patterns observed across subgroups. A functional marker, such as PPi, may therefore capture reduced ENPP1 function more directly than *ENPP1* genotype alone. This interpretation supports the possibility that reduced ENPP1 function contributes to the pathophysiology of OPLL, while also indicating that its effects are not simply expressed as the extent or distribution of ectopic ossification. Rather, the ultimate phenotype of ectopic ossification is likely shaped by interactions among aging, metabolic factors, mechanical stress, and other bone and mineral regulatory pathways. These findings are consistent with the current pathophysiological understanding that OPLL is a multifactorial disorder with a complex pathogenesis.

As one potential acquired cause of reduced ENPP1 function, we investigated the possible involvement of anti-ENPP1 autoantibodies using a LIPS assay with ENPP1-NanoLuc and NanoLuc-ENPP1 constructs; however, no apparent autoantibodies were detected in either system. Notably, no apparent anti-ENPP1 autoantibodies were detected even among the 8 variant-negative patients with plasma PPi levels below the median of the original cohort, a subgroup of particular interest when considering an acquired mechanism. This finding suggests that, at least in the patients with OPLL examined in this study, anti-ENPP1 autoantibodies are unlikely to represent a cause of reduced ENPP1 function. Accordingly, in patients without *ENPP1* variants who nevertheless exhibited low plasma PPi levels, acquired factors other than autoantibodies may contribute to the observed ENPP1 dysfunction.

Previous studies have shown that inflammation and oxidative stress can affect ENPP1 expression and function. Krzyzanowska et al. demonstrated that TNF-α stimulation suppresses ENPP1 expression in human and bovine intervertebral disc cells via the NF-κB pathway.^19^ Ferreira et al. reported that IL-1β, a representative proinflammatory cytokine, reduces both ENPP1 expression and enzymatic activity in human mesenchymal stem cells.^20^ In addition, Yuan et al. showed that oxidative stress suppresses ENPP1 expression in cartilaginous endplate chondrocytes.^21^ Taken together, these findings suggest that inflammatory conditions and oxidative stress may affect ENPP1 function as acquired mechanisms and thereby contribute to the pathogenesis of OPLL. In the present exploratory analyses, serum CRP levels did not differ according to *ENPP1* variant status or plasma PPi group and were not correlated with plasma PPi levels. In addition, serum CRP levels were not significantly correlated with radiographic ossification indices in the overall cohort or in analyses stratified by *ENPP1* variant status. Thus, the present data did not demonstrate an association between systemic inflammation, as reflected by CRP, and reduced ENPP1 function or radiographic ossification burden. However, CRP is a nonspecific marker of systemic inflammation and may not reflect local inflammatory or oxidative processes within spinal ligaments or intervertebral disc tissues. Furthermore, CRP and plasma PPi were not measured concurrently in all patients. Therefore, a contribution of local inflammation or oxidative stress to ENPP1 dysfunction and the pathogenesis of OPLL cannot be excluded.

Recent progress has been made in the development of BMN 401 (formerly INZ-701), a soluble, non-bone-targeted ENPP1–Fc fusion protein, as ENPP1 replacement therapy for ENPP1 deficiency, and therapeutic strategies aimed at restoring ENPP1 activity have entered clinical testing.^22^^,^^23^ By contrast, the suppression of spinal hyperostosis and amelioration of enthesopathy in the preclinical OPLL mouse model were achieved using a bone-targeted ENPP1 enzyme.^14^ These agents should therefore not be regarded as interchangeable, and whether bone targeting is required for efficacy against skeletal and entheseal lesions, such as OPLL and DISH, remains to be determined. Publicly reported results from the phase 3 ENERGY 3 trial in children with ENPP1 deficiency showed that BMN 401 significantly increased plasma PPi but did not meet the radiographic co-primary endpoint for rickets severity.^24^ However, the implications of this result for OPLL and DISH are limited because the skeletal manifestations are mechanistically distinct. ARHR2 is an under-mineralization disorder driven primarily by FGF23-mediated hypophosphatemia, whereas OPLL and DISH are characterized by pathological ectopic ossification. Raising PPi does not directly correct the principal driver of skeletal disease in ARHR2, whereas PPi restoration could be mechanistically relevant to suppressing ectopic ossification because PPi inhibits hydroxyapatite nucleation and crystal growth. Therefore, the radiographic result in ARHR2 neither confirms nor refutes the therapeutic hypothesis in OPLL or DISH.

Taken together, these findings support PPi restoration as a therapeutic hypothesis warranting direct testing in patients with OPLL or DISH who show functional ENPP1 impairment. However, its efficacy in humans remains unproven and should be evaluated in the relevant population using an appropriately targeted agent.

This study has several limitations. First, this was a secondary, exploratory analysis of a previously reported single-center cohort, and its cross-sectional, association-based design precludes inference of causality or directionality. Second, the cohort was small (*n* = 50), particularly the *ENPP1* variant-positive group (*n* = 7), limiting statistical power and rendering subgroup estimates unstable. Third, multiple comparisons were performed without adjustment for multiplicity. Accordingly, the associations between *ENPP1* variant status and serum FGF23 levels and between lower plasma PPi levels and DISH should be considered hypothesis-generating and would not remain statistically significant after conventional correction for multiple comparisons. Fourth, several findings do not support a simple relationship between reduced ENPP1 function and clinical or radiographic severity, including the lower prevalence of gait disturbance and muscle weakness among variant carriers, the absence of differences in radiographic ossification indices, and the fact that the median FGF23 levels in both groups were within the reference range. Finally, because the participants were patients evaluated for surgical treatment, the generalizability of these findings to the broader population of patients with OPLL or DISH is limited. Adequately powered studies with prespecified endpoints and appropriate control for multiplicity are required to validate these findings.

## Supplementary Material

Supplementary_Methods_ziag157

## Data Availability

All data generated or used during the study are available from the corresponding author and first author upon reasonable request.
