# Supplementary material for: Involvement of reduced ENPP1 function in the pathogenesis of ossification of the posterior longitudinal ligament
Source: JBMR Plus. 2026 Sep 17;10(11):ziag157. doi: 10.1093/jbmrpl/ziag157 (PMC13630564; doi:10.1093/jbmrpl/ziag157)
Supplement: Supplementary file 1 [file supplementary_methods_ziag157.pdf]

Detection of anti-ENPP1 autoantibodies by LIPS assay

A luciferase immunoprecipitation system (LIPS) assay was performed using serum samples from 20 of the 50 patients with OPLL who underwent genetic analysis and 9 additional patients with OPLL enrolled later. ENPP1 cDNA was amplified by PCR using Human Universal QUICK-Clone™ cDNA II (Clontech, Mountain View, CA, USA) as a template. NanoLuc cDNA, encoding an engineered luciferase, was amplified using the pNL1.1 vector (Promega, Madison, WI, USA) as a template. The PCR products were verified by agarose gel electrophoresis and purified using the QIAquick Gel Extraction Kit (Qiagen, Hilden, Germany). The purified ENPP1 and NanoLuc cDNA fragments were cloned into the pcDNA3.1(+) vector (Invitrogen, Carlsbad, CA, USA), which had been pre-digested with BamHI and EcoRI, using the In-Fusion HD Cloning Kit (Clontech). Two full-length ENPP1 constructs fused with NanoLuc luciferase at either the C terminus or N terminus, ENPP1-NanoLuc and NanoLuc-ENPP1, respectively, were generated. The resulting vectors were transformed into *Escherichia coli* DH5α, and plasmids were isolated using the QIAGEN Plasmid Mini Kit (Qiagen). Sanger sequencing was performed to confirm the correct insertion and sequence accuracy. After sequence verification, high-purity plasmids were prepared using the QIAGEN Plasmid Maxi Kit (Qiagen). Each plasmid was transfected into

COS-7 cells using Lipofectamine 3000 Reagent (Invitrogen), and crude cell lysates were collected after 48 hours of culture.

For the LIPS assay, 1 μL of serum was mixed with 49 μL of buffer A, consisting of 20 mM Tris-HCl pH 7.5, 150 mM NaCl, 5 mM $MgCl_2$, and 1% Triton X-100. Protein A/G magnetic beads (Dynabeads Protein A and G, Invitrogen) were then added, and the mixture was incubated for 10 min at room temperature. As negative controls, serum samples from two patients each with tumor-induced osteomalacia, Cushing's disease, hypophosphatasia, Graves' disease, and hypoparathyroidism were used, for a total of 10 disease-control samples. For baseline luminescence measurements, a no-serum buffer-only condition was included. A commercially available anti-ENPP1 antibody (Biorbyt, Cambridge, UK) was used as an assay positive control.

After the beads were washed with buffer A, 16 μL of cell lysate expressing either ENPP1-NanoLuc or NanoLuc-ENPP1 was diluted with buffer A to a final volume of 100 μL and incubated with the beads for 1 hour at room temperature. The beads were then washed three times with buffer A and finally resuspended in 50 μL of buffer A.

For luminescence measurement, 50 μL of Nano-Glo Luciferase Assay Substrate (Promega) was added, and the samples were dispensed into a 96-well microplate.

Luminescence was measured using a GloMax Discovery Multimode Microplate Reader

(Promega), and the signals were analyzed as relative luminescence units.

Supplementary Table 1. Characteristics of all 50 patients with OPLL

No.	*ENPP1* variant	Age/Sex	Ht/Wt cm/kg	BMI	Family history	Age at diagnosis	HbA1c %	Pi mg/dL	eGFR mL/min /1.73m^2	ALP U/L	iPTH pg/mL	FGF23 pg/mL	PPi nM	DISH	OA-index	OP-index	OY-index	OS-index	No. of surgeries	Age at first surgery
1	c.802T>C p.Y268H	65/F	161/60	23.1	Yes	68	6.2	4.1	77.4	114	52	45.4	1915	No	13	9	6	28	1	65
2	c.802T>C p.Y268H	82/M	166/78	28.3	Yes	35	5.4	3.1	45.2	46	58	35.8	1495	Yes	0	6	11	17	0	
3	c.1352A>G p.Y451C	69/M	161/55	21.2	No	73	5.6	3.8	43.6	75	95	49.8	1719	No	17	5	3	25	1	73
4	c.2089G>A p.V697M	46/M	167/63	22.6	No	57	5.7	3	58.8	198	125	41.9	1370	Yes	3	5	4	12	1	57
5	c.2335A>C p.T779P	45/M	187/135	38.6	No	66	5.3	3.2	78.2	62	22	46.2	1402	Yes	2	3	2	7	1	78
6	c.2335A>C p.T779P	71/M	162/97	37.0	No	80	6.1	2.8	57.6	82	91	78.2	957	Yes	14	12	3	29	1	82
7	c.2335A>C p.T779P	82/M	164/62	23.1	No	63	5.3	3.6	55.4	87	34	102	1477	Yes	9	3	13	25	1	63
8		73/F	148/65	29.7	No	84	8.3	3.7	62.8	30	24	47.4	1987	No	15	4	1	20	1	84
9		74/M	168/72	25.5	No	50	6.1	2.7	47.5	68	51	52.7	2240	Yes	3	5	0	8	1	52
10		75/F	146/35	16.4	No	60	7.3	3.1	92.7	73	68	25.6	1639	Yes	7	2	0	9	1	61
11		56/M	170/79	27.3	No	47	6	3.9	89.3	109	125	42.4	2332	Yes	18	3	12	33	1	56
12		49/M	165/105	38.6	No	61	7.7	3.3	77.8	75	51	61.5	2340	No	18	4	1	23	1	63
13		60/F	140/57	25.7	No	49	5.6	3.3	57.6	79	39	31.8	2611	No	13	10	12	35	1	49
14		76/M	160/67	26.2	No	43	5.7	3.2	87.1	66	52	36.8	1998	No	1	5	11	17	1	43
15		56/F	160/75	29.3	No	73	6.1	3.8	79	73	55	31.1	2372	No	2	15	11	28	2	73
16		59/M	168/70	24.8	Yes	50	7.5	3.5	65.2	80	30	27.3	2551	No	20	11	6	37	1	74
17		75/F	144/63	30.4	No	74	5.8	3.7	48.4	80	99	36.9	2705	No	12	17	5	34	2	74
18		37/F	173/54	18.0	No	60	5.2	3.2	67.7	42	90	20.7	1613	No	0	0	4	4	2	61
19		73/M	158/54	21.6	Yes	46	5.1	3.7	68.5	70	47	33.9	1534	Yes	20	9	5	34	1	48

20	57/M	177/62	19.8	No	54	5.9	3.2	100.8	79	39	17.4	1768	No	14	2	2	18	1	56
21	78/F	159/56	22.2	No	46	6.0	3.7	63.6	99	51	59	1621	No	13	36	11	60	3	47
22	82/F	149/62	27.9	No	80	6.3	3.5	58	63	48	44.4	1693	Yes	9	3	0	12	1	80
23	63/M	173/81	27.1	No	39	5.7	3.7	73.4	79	33	37.3	2364	Yes	16	19	14	49	2	39
24	84/M	152/56	24.2	No	59	5.7	3	72.4	73	34	37.9	1524	Yes	1	3	2	6	1	60
25	51/M	161/67	25.8	No	72	7.7	2.8	88.6	65	29	30.5	1475	No	8	4	3	15	1	74
26	61/F	162/73	27.8	No	69	6.2	3.3	58.6	81	81	54.4	1357	Yes	19	9	3	31	2	70
27	46/M	171/110	37.6	No	46	5.6	2.2	78.2	139	67	27.1	1563	Yes	9	3	7	19	2	46
28	63/M	170/83	28.7	No	50	6.1	3	59	42	33	28.5	1692	Yes	4	1	7	12	1	63
29	49/M	175/91	29.7	No	75	5.8	4.2	89.6	78	64	37.5	2097	Yes	1	4	0	5	1	76
30	49/M	176/100	32.3	No	59	5.6	4.1	93.8	128	70	46.4	850	No	8	4	3	15	1	60
31	63/F	153/56	23.9	Yes	56	5.3	3.2	89.7	68	77	32.7	1732	No	0	11	9	20	1	56
32	80/F	155/82	34.1	No	77	5.9	3.1	74.0	91	30	47.3	1849	Yes	16	4	0	20	1	77
33	72/M	163/66	24.8	No	59	5.4	3	56.9	127	47	40.6	1621	Yes	0	8	0	8	1	59
34	73/M	158/58	23.2	No	52	6.4	2.8	62.8	74	49	23	3012	Yes	16	11	5	32	1	53
35	46/M	174/76	25.1	No	80	5.6	3	109.1	80	24	29.1	1878	No	14	3	3	20	1	82
36	51/M	173/78	26.1	No	47	5.3	1.9	52	78	98	86	1261	No	22	13	13	48	1	51
37	80/M	169/71	24.9	No	62	6.1	3.0	39.5	69	44	42.5	1523	Yes	10	3	3	16	1	66
38	56/M	170/84	29.1	No	63	6.0	2.8	65	143	132	35.6	1209	Yes	11	11	11	33	1	53
39	57/M	180/110	34.0	No	83	4.9	3.3	73.9	175	38	41.5	1466	No	16	7	2	25	1	83
40	78/M	170/70	24.2	No	78	7.1	3	51.3	165	70	52.2	1226	Yes	30	10	2	42	1	79
41	67/F	152/78	33.8	No	72	5.7	3.1	65.7	97	65	26.4	1979	Yes	1	3	2	6	1	75
42	83/M	163/63	23.7	No	75	6.5	3.6	39.3	93	23	36.9	1554	Yes	13	6	2	21	1	76

43	79/M	161/60	23.1	No	61	5.0	3.2	74	113	54	18.8	1604	Yes	20	15	2	37	1	65
44	73/M	159/53	21.0	No	68	5.1	2.8	64.3	97	142	28.5	1454	No	5	3	6	14	1	68
45	76/M	167/62	22.2	No	73	6.1	3.3	79	102	58	35.8	1792	Yes	4	12	3	19	1	75
46	71/M	165/81	29.8	No	69	5.8	2.7	62.5	103	56	34.6	1413	Yes	23	10	3	36	1	71
47	60/M	170/66	22.8	No	44	5.6	2.7	54	51	76	45.1	1719	No	1	2	0	3	2	45
48	86/M	156/62	25.5	No	57	5.4	4.1	65.4	67	28	32.3	1492	Yes	15	9	2	26	1	86
49	78/F	148/55	25.1	No	73	6.1	5.1	47.2	89	51	35.5	2003	Yes	12	5	1	18	1	78
50	52/M	172/78	26.4	No	38	6.0	3.3	76.5	101	25	14	2197	No	0	3	0	3	1	38

Ht, height; Wt, weight; BMI, body mass index; Pi, inorganic phosphate; eGFR, estimated glomerular filtration rate; ALP, alkaline phosphatase; iPTH, intact parathyroid hormone; FGF23, fibroblast growth factor 23; PPi, inorganic pyrophosphate; DISH, diffuse idiopathic skeletal hyperostosis; OA-index, ossification of the anterior longitudinal ligament index; OP-index, ossification of the posterior longitudinal ligament index; OY-index, ossification of the yellow ligament index; OS-index, the total ossification index.
